# Light conditions influence optic nerve OCT angiography parameter in healthy subjects with neutral pupils

**DOI:** 10.1038/s41598-023-36069-5

**Published:** 2023-06-06

**Authors:** Pieter Nelis, A. Nieweler, V. Brücher, N. Eter, M. Ten Tusscher, M. Alnawaiseh

**Affiliations:** 1 Department of Ophthalmology, University of Muenster Medical Center, Albert-Schweitzer-Campus 1, Building D15, 48149 Muenster, Germany; 2 Department of Ophthalmology, Vrije Universiteit Brussel, Brussels, Belgium; 3 Department of Ophthalmology, Helios Augenklinik Berlin-Buch, Berlin-Buch, Germany

**Keywords:** Medical research, Diagnosis

## Abstract

Optical coherence tomography angiography measurements are influenced by a range of environmental factors as blood pressure and physical fitness. The present study aimed to evaluate the effects of light and dark exposure in eyes with neutral and mydriatic pupils on vessel density in the macular and optic nerve head regions, as measured using optical coherence tomography angiography (OCTA). 55 eyes of 55 healthy volunteers (28 patients with neutral pupils; 27.18 ± 4.33 years) were examined using a high-speed and high-resolution spectral-domain OCT XR Avanti system with a split-spectrum amplitude de-correlation angiography algorithm. OCTA imaging was performed after dark adaptation and after exposure to light. The vessel density data of the superficial and deep retinal macular and optic nerve head region OCT-angiogram were analyzed for these two light conditions. Through Bonferroni correction for multiple testing, the p- value was adapted from 0.05 to 0.017. In eyes with neutral pupils, a significant increase was found in the capillary region of the optic nerve head region (p = 0.002), comparing dark- and light-adaptation. In the macular region of eyes with neutral (p = 0.718) and mydriatic pupils (p = 0.043), no significant differences were observed, as were any in the optic nerve head region of the mydriatic eyes (p = 0.797). This observation suggests that light conditions could be a possible factor influencing OCTA measurements. After dark exposure, vessel density data were significantly different between eyes with neutral and mydriatic pupils (nerve head region: p < 0.0001, superficial macula: p < 0.0001, deep macula: p = 0.0025). These data warn for the effect of mydriatic drops on vessel density measurements.

## Introduction

The influence of light conditions on the retina is well documented. Electrophysiological tests as full-field electroretinogram describe this response as a retinal depolarization after light stimulation^1^. Retinal polarization changes lead to differences in energy demands and blood flow adaptation^2^. This complex process is called neurovascular coupling^3–6^. Distinct influences, e.g. flicker vs. continuous light, and lighting conditions such as scotopic and photopic illumination require a change in blood flow. This is accomplished by a group of cells of both vascular and neural origin, referred to as the neurovascular unit^7^.

Neurovascular coupling is not exclusively found in the retina. The brain, using 20% of the body’s energy at rest, has extensive neurovascular coupling mechanisms, which are needed to adapt to changing stimuli^3,8^. Neurovascular coupling is known to be disrupted in different conditions such as ischemic stroke, Alzheimer’s disease, or hypertension. Therapeutic strategies aiming to improve retinal or cerebral blood flow in diseases like ischemic stroke or hypertension require a precise understanding of this mechanism^3,9^.

As the retina is the most accessible part of the brain and the retinal vasculature can be visualized with non-invasive techniques, the eye is well suited to study neurovascular coupling^10,11^. Despite this, there have been few studies to date investigating neurovascular coupling in the eye^12^. Most of the published data draw conclusions about flow changes based on vascular caliber variations^13,14^.

OCT angiography (OCTA) is a non-invasive imaging technique that enables visualization and quantification of blood flow in the retina and in the optic nerve head (ONH). The technology has proven to be very valuable for the diagnosis and follow up of various ocular and systemic vascular diseases. As OCTA is fast and easily repeatable, it is well accepted by patients and clinicians. Since the introduction of OCTA research interest in the quantification of blood flow in the optic nerve and macular region is growing steadily^15–17^.

The present study evaluates the effects of light and dark exposure on vessel density (VD) in the retina and ONH using OCTA. The aim was to determine the feasibility of monitoring neurovascular coupling. The results were obtained in eyes with neutral pupils as well as in mydriasis in two different cohorts to exclude bias induced by post-dark adaptation pupillary constriction.

## Materials and methods

### Subjects

Fifty-five healthy subjects were included in this study. All investigations were performed in accordance with the declaration of Helsinki and written informed consent was obtained from all participants before imaging. The study was approved by the local ethics committee of the medical association of Westfalen-Lippe and the Westphalian Wilhelms-University of Muenster. Before imaging, a complete ophthalmic examination was performed including best corrected visual acuity, measurement of intraocular pressure (IOP), slit lamp biomicroscopy and funduscopy of the macula and ONH.

Inclusion criteria were age > 18 years and in good health, defined as the absence of cardiovascular diseases, high blood pressure, diabetes, sleep apnea and other systemic diseases^18–20^. Exclusion criteria were ocular pathologies on examination or in the patient’s history, and spherical equivalents outside the range of − 3.5 and + 3.5. Patients with any history of ophthalmic surgery or treatment, lens opacities, corneal opacities or refractive surgery were also excluded.

### Study protocol

**Figure 1:**
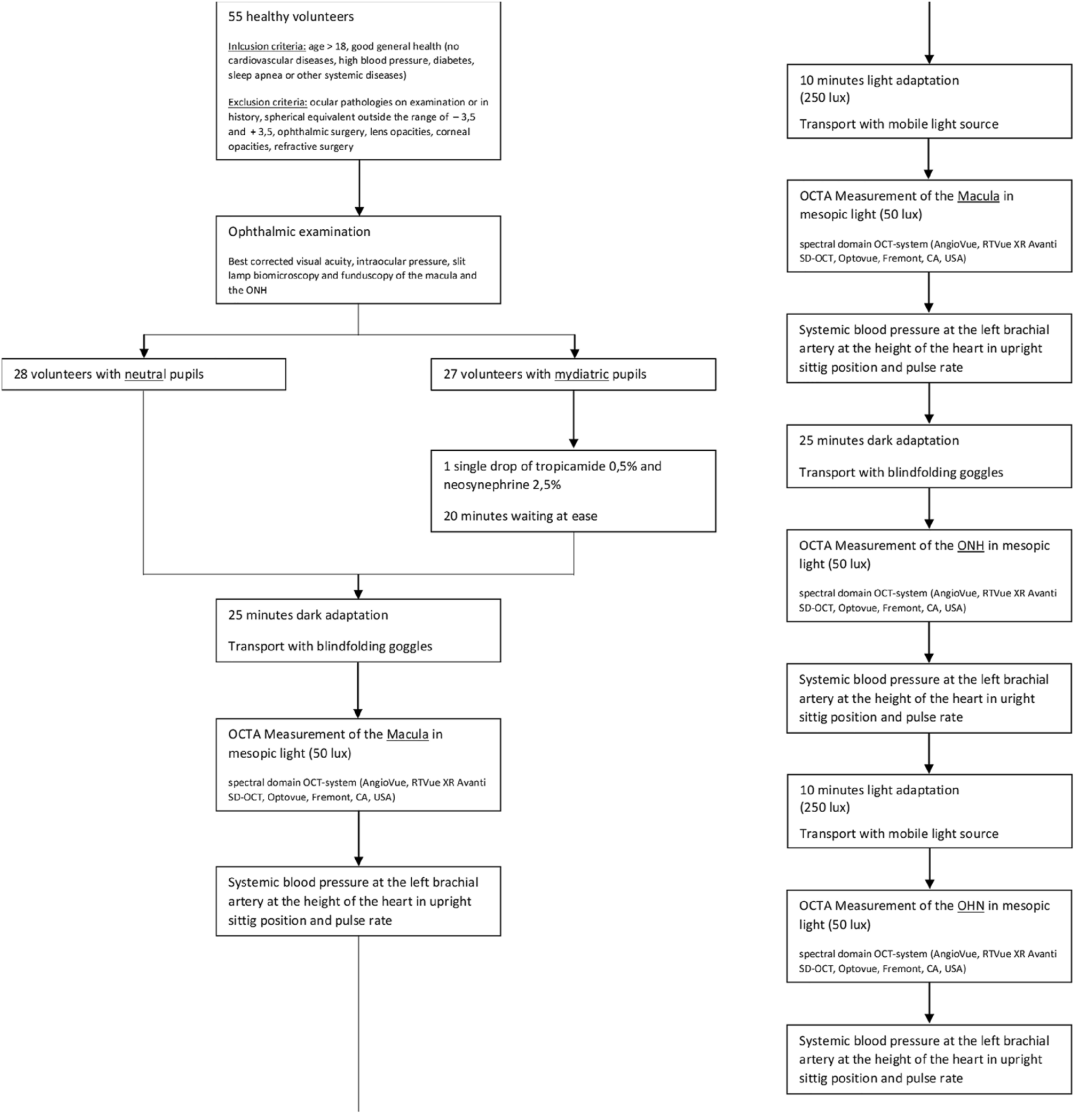
Flow chart of the study protocol.

Subjects were randomly assigned to one of two groups (neutral or mydriatic pupils). Patients were not asked to undergo the cycles of dark and light adaptation with both neutral and mydriatic pupils to improve compliance (Fig. 1).

Before OCTA imaging of the ONH and macular regions, the subjects were put at ease. Immediately after performing each OCTA measurement, systemic blood pressure and the pulse rate were determined at the left brachial artery at the height of the heart with the subject in an upright sitting position.

After dark adaptation for 25 min OCTA measurements were performed in mesopic light conditions (indirect surrounding light, 50 lx)^21^. The participants remained in dark adaptation during transport from the darkened room to the OCTA device by way of blindfold goggles. Subsequently, the subjects were light adapted using a 250-lx light box for 10 min followed by OCT-A measurements in mesopic light conditions. All images were recorded in the right eye by an experienced operator under the same standardized mesopic lighting conditions in the same location. Measurements of the macular and ONH region were performed in separate cycles of dark and light adaptation. The group of subjects with neutral pupils did not receive eye drops. The group of subjects with pupils in mydriasis underwent pupil dilation using 1 single drop of a tropicamide 0,5% and neosynephrin 2.5% mixture. 20 min after instillation dark adaptation was initiated.

### OCT angiography

OCTA technology has been described in detail previously^17,22–24^. Imaging was conducted with a spectral domain OCT-system (AngioVue, RTVue XR Avanti SD-OCT, Optovue, Fremont, CA, USA). The device delivered volumetric scans of 304 × 304 A-scans at 70,000 A-scans per second using a light source at 840 nm. Two consecutive B-scans covering the central 3 × 3 mm^2^ field of the macula and the 4.5 × 4.5 mm^2^ ONH field were performed to compute inter-B-scan decorrelation with the SSADA algorithm. The software version used in this study included the eye tracking function (DualTrac Motion Correction, Optovue, Fremont, CA, USA).

**Figure 2:**
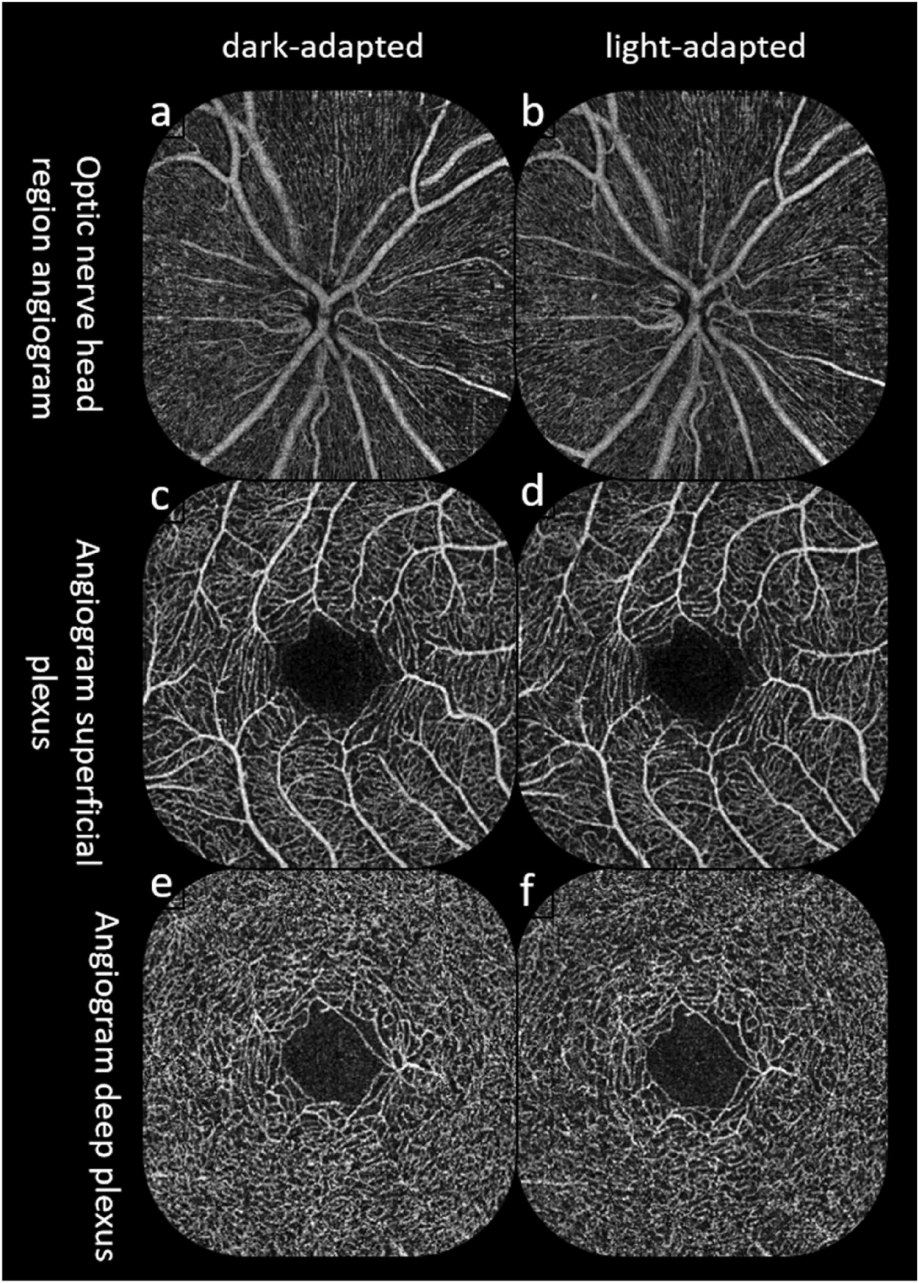
Exemplary angiograms from one participant. Left column shows angiograms of optic nerve head region (**a**), macular superficial plexus (**c**) and macular deep plexus (**e**) after dark adaptation. The right column shows angiograms of optic nerve head region (**b**), macular superficial plexus (**d**) and macular deep plexus (**f**) after light adaptation.

VD was then calculated by first extracting a binary image of the vessels from the grayscale OCTA en-face image, and then computing the percentage of pixels of vessels in the defined sectors or in the entire en-face image based on the binary image^23^. After checking for proper segmentation, VD data were evaluated in the superficial plexus (VDsM) and the deep plexus (VDdM) of the central macula and in the radial peripapillary capillary layer of the peripapillary region (VDrpcPP) (Fig. 2). For the VDprcPP, two options are available in the analysis menu: “capillary” and “all”. We opted for the capillary values, thus excluding the large vessels. These analyses were performed with proprietary software of Optovue (ReVue 2017.1.0.151). Images were excluded, when quality index was below 6 or when uncorrectable segmentation errors were present in the quantifiable region^24^.

### Statistical analysis

Microsoft Excel 2010 was used for data management. Statistical analyses were performed using Prism 7.02 (GraphPad Software nc., La Jolla, USA). The sample size was calculated based on a previous study with a detected effect size (ES) = 0.73, revealing that a sample size of 23 would yield a statistical power of (1-beta) = 0.96 at alpha = 0.05^14,25^. OCTA data were tested for normal distribution using the D'Agostino-Pearson normality test (omnibus K2 test). Data presented as mean ± SD and median [interquartile range]. Differences between measurements were determined using the two-sided paired t-test for normal distributed data or Wilcoxon paired t-test in the case of non-normal distributed data. To compare baseline OCTA data between eyes with neutral and mydriatic pupils, the two-sided unpaired t-test for normal distributed data or Mann–Whitney t-test in the case of non-normal distributed data were instrumented. To compare the distribution of baseline OCTA data between eyes with neutral and mydriatic pupils, the two-sample Kolmogorov–Smirnov-test. For the t-tests, p-value was adapted through Bonferroni correction for multiple testing from 0.05 to 0.017. For the correlation analysis, p- value was adapted through Bonferroni correction for multiple testing from 0.05 to 0.01.

## Results

55 healthy volunteers (neutral group [n = 28]: 26.88 years ± 4.91; mydriasis group [n = 27]: 27.48 years ± 3.69) were included prospectively in this study. Systemic, cardiovascular parameters (heart rate and blood pressure) and image quality index were not significantly different between eyes with neutral and mydriatic pupils. Further characteristics of the study population are summarized in Table 1.

**Table 1:** Population characteristics and general study parameters: sex, age (years), spherical equivalent (diopters), intraocular pressure (mmHg), quality parameters of macular and optic disc angiograms.

	Neutral	Mydriasis	
Sex	F 19–M 9	F 17–M 10	
Age (years)	26.88 ± 4.91	27.48 ± 3.69	0.03
Spherical equivalent (diopter)	− 0.68 ± 1.07	− 0.64 ± 1.00	0.52
Intra-ocular pressure (mmHg)	16.50 ± 2.06	15.81 ± 3.38	0.92
Quality index of peripapillary region	7.78 ± 0.85	7.82 ± 0.88	> 1.00
Quality index of macular region	7.82 ± 0.68	7.93 ± 0.86	0.704

Data presented as mean ± SD.

P-values were determined using Kolmogorov–Smirnov test.

In the group with neutral pupils, after dark adaptation, 1 optic disc image and 1 macular image, and after light adaptation, 3 optic disc images were excluded due to a low quality index. In the group with mydriatic pupils, after dark adaptation, 1 macular image, after light adaptation, 1 optic disc images and 1 macular image were excluded due to a low quality index. No projection artefacts were observed in the deep retinal plexus.

**Figure 3:**
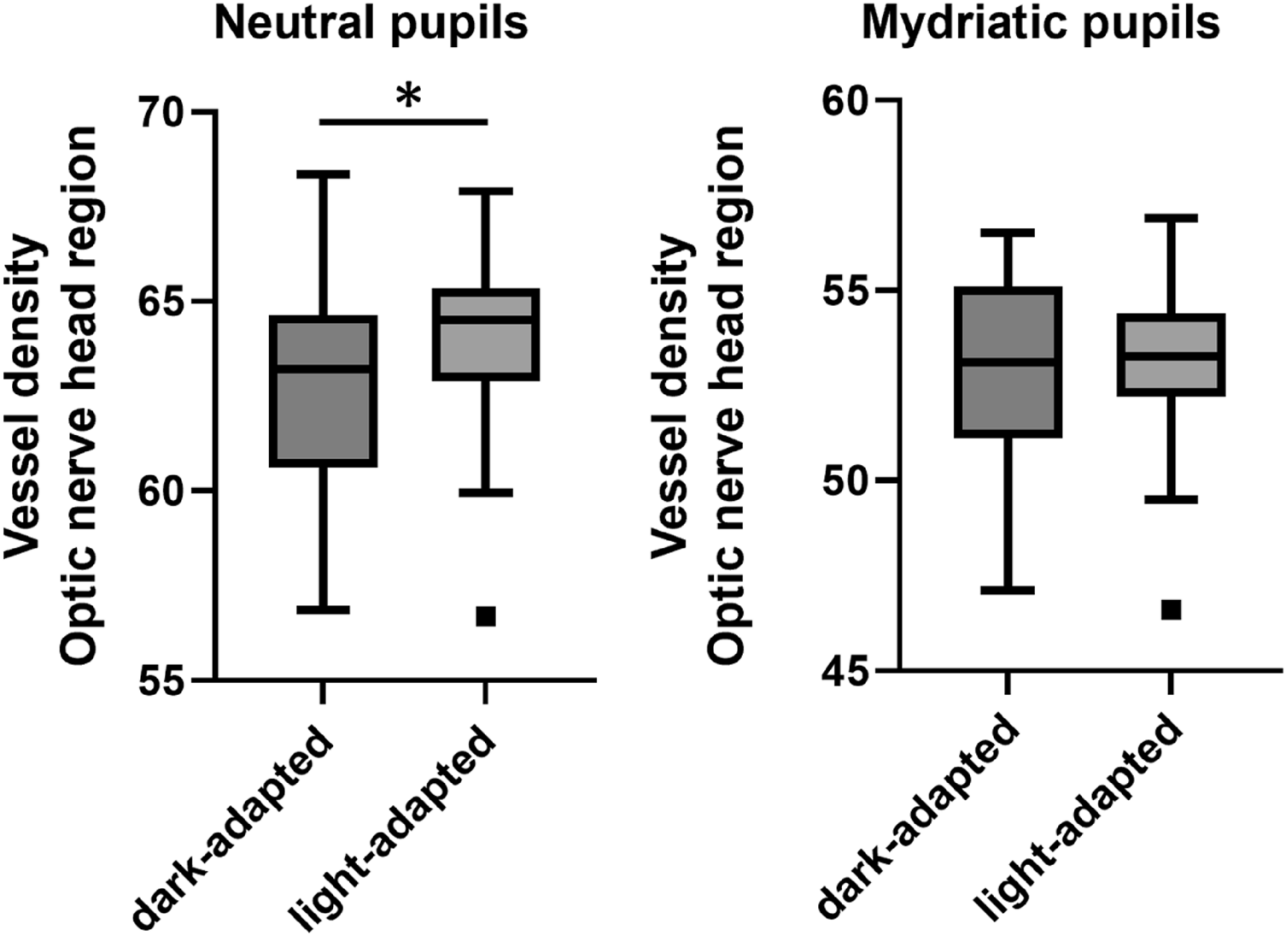
Tukey plot of vessel density of radial peripapillary capillary layer of peripapillary area after dark and light adaptation in patient with neutral and mydriatic pupils. *Statistically significant.

In eyes with neutral pupils, a significant increase in VD was found in the RPC layer of the peripapillary region between dark- and light-adapted states. (Fig. 3) In the macular region of eyes with neutral and mydriatic pupils, no significant differences in VD were observed, as were any in the RPC layer of the peripapillary region of the mydriatic eyes. (Table 2). Comparing baseline OCTA data between eyes with neutral and mydriatic pupils, statistically significant differences were found: VDrpcPP: p < 0.0001, VDsM: p < 0.0001, VDdM: p = 0.0025. Comparing differences in distribution of OCTA baseline data between eyes with neutral and mydriatic pupils, statistical differences were found: VDrpcPP: < 0.0001, VDsM: < 0.0001, VDdM: 0.0017.

**Table 2:** Results of analysis of macular and ONH region vessel density in eyes with neutral and mydriatic pupils and in two light conditions: dark-adapted and light-adapted states.

	Neutral			Mydriasis		
	Dark-adapted	Light-adapted	p-value	Dark-adapted	Light-adapted	p-value
VD of radial peripapillary capillary layer of peripapillary area	62.55 ± 2.51	64.07 ± 2.73	0.002*	52.90 ± 2.37	52.97 ± 2.21	0.797^#^
	63.20 [60.77–64.53]	64.40 [62.53–65.80]		53.10 [51.10–55.10]	53.25 [52.20–54.40]	
VD of superficial layer of macular area	53.48 ± 3.75	53.88 ± 4.39	0.718*	47.22 ± 3.60	48.60 ± 2.86	0.043*
	54.50 [51.40–56.40]	55.12 [51.20–57.31]		47.20 [45.98–49.25]	48.80 [46.55–50.75]	
VD of deep layer of macular area	57.40 ± 6.14	58.19 ± 5.36	0.312*	52.14 ± 6.07	52.14 ± 4.99	0.939*
	59.70 [51.70–62.61]	61.09 [52.35–62.63]		52.55 [50.43–56.03]	52.80 [49.85–55.25]	

Significant values are in bold.

Data presented as mean ± SD and median [interquartile range]. Differences between groups were calculated using the Wilcoxon test for non-normal distributed data (*) or paired t-test for normal distributed data (^#^). *VD* vessel density.

Differences between groups were calculated using the *Wilcoxon test for non-normal distributed data or ^#^paired t-test for normal distributed data.

A correlation analysis was performed between baseline OCTA data and several other parameters: age, intraocular tension, spherical equivalent, mean arterial pressure and heart rate. This did not reveal significant correlations. The correlation between spherical equivalent and VDdM in eyes with neutral pupils almost reached statistical significance (spearman r = 0.481, p = 0.011). The correlation between the macular quality index and VDsM in eyes with mydriatic pupils almost reached statistical significance (spearman r = 0.487, p = 0.0116).

## Discussion

In this study, macular and ONH region vessel density data were analyzed in eyes with neutral and mydriatic pupils and in two light conditions: dark-adapted and light-adapted states. In eyes with neutral pupils, a significant change was found in the RPC layer of the peripapillary region between light- and dark-adapted states. In the macular region of eyes with neutral and mydriatic pupils, no significant differences were observed, as were any in the RPC layer of the peripapillary region of the mydriatic eyes. After dark exposure, vessel density data were significantly different between eyes with neutral and mydriatic pupils.

OCT-A is a much used but still relatively novel non-contact imaging technique, which can be performed easily and fast. It enables the visualization of retinal and choroidal vessels and vessels in the ONH region without the need for intravenously injected dye. OCTA therefore attracted considerable ophthalmological clinical research interest over the last 5 years^17^. Moreover, OCTA enables quantitative analysis of ocular flow: the repeatability of vessel density data using OCT-A has been analyzed in healthy subjects and in patients with various ocular and systemic diseases as well as in different animal models^15–17,22,23,26,27^. Consequently, an increasing amount of systemic and environmental factors have been shown to influence OCTA measurements. Neurovascular coupling effects, like light and dark adaptation, on OCTA have been studied only in eyes with neutral pupils^28^. Since pupil diameter is known to influence optic nerve OCT measurement, a parallel evaluation in eyes with neutral and mydriatic pupils seems reasonable^29^. This pilot study evaluates changes in vessel density, measured by OCTA, in response to dark and light exposure in both eyes with neutral and mydriatic pupils.

The retina is supplied by the central retinal artery and the short posterior ciliary arteries. The macular retinal circulation can be divided into four layers: radial peripapillary capillary plexus, the superficial vascular plexus, the intermediate capillary plexus and the deep capillary plexus^30^. The last two layers have a spider-like appearance with small feeding vessels^31,32^. In the peripapillary region, the radial peripapillary capillary network supplies the unmyelinated nerve fibres^33,34^. Roy and Sherrington hypothesized more than 100 years ago that the brain could vary the vascular supply in response to variations in functional activity^35^. About a century later different studies demonstrated similar phenomena for the ONH region and major retinal vessels^12,36^. To compensate for elevated oxygen consumption of photoreceptors in the dark, ocular perfusion increases^7,10^. Experiments on neurovascular coupling revealed two possible mechanisms which might act in synergy. (i) Local metabolic feedback systems, in which neural activity leads to energy demand and vasodilation, are the most traditional way to explain NVC^9^. (ii) Neurons are postulated to increase local blood flow directly or via astrocytes releasing vasoactive agents. This glutamate-mediated process is called functional hyperemia^5,37,38^.

Literature on the effects of dark adaptation on flow is not conclusive. In 1983 Feke et al. used laser Doppler techniques to demonstrate a retinal blood flow increase after the transition from light to dark^39^. Using bidirectional laser doppler velocimetry, Riva et al. showed an increase in diameter and blood flow velocity in major veins, taking place 5 min into dark adaptation^40^. However, newer studies using static registration methods as scanning laser ophthalmoscopy and retinal vessel analyzers did not find any significant increase in retinal branch diameter during dark adaptation^41,42^. Even more, a more recent study of Riva et al. using laser Doppler flow measurements found no change in blood flow during dark adaptation^43^. The present results of the peripapillary region in eyes with neutral pupils contradict the findings of Feke et al. and the earlier results of Riva et al. Of note, Riva et al. and Feke et al. measure blood flow of large vessels directly around the optic nerve, whereas our study only analyses the capillaries of the peripapillary region, disregarding the larger vessels^39,40^.

Dark and light stimulation has often been combined with flicker stimuli. Flicker-induced arterial and venular vasodilation have been more consistently reported in literature, especially in the inner retina, more susceptible to these stimuli. Due to technological limitations, these studies focused on the larger vessels. OCTA provides a higher axial resolution, enabling the visualization of capillaries, in combination with a depth-resolution. Instrumenting these OCTA features, Nesper et al. aimed to include light and dark adaptation, as well as flicker-stimuli. Nesper et al. reported a statistically significant increase, resp. decrease in the superficial and intermediate layers following the transition from dark to ambient light. In the deep layer they did not find a significant difference (p < 0.05)^28^. Additionally, they also found a significant increase in superficial layer during flicker stimulation compared to dark adaptation. They attributed this to constriction of the deep and intermediate layers and a large vessel dilation response in the superficial layer. In the macular region of eyes with neutral pupils, we do not find similar results as in Nesper et al. This incongruence might be explained by the different segmentation strategies used: Nesper and colleagues used a three-slab segmentation, whereas we used the more widespread 2-slab segmentation. The possible advantage of the latter is the arguably fewer segmentation errors since only one segmentation border must be checked. A major disadvantage is a lower sensitivity to layer-specific reactions to stimuli^44,45^. The three plexuses are differentially regulated. Kornfield and Newman have showed that even between the superficial, intermediate, and deep layers divergence in effects is possible. Duan et al. even found different effects to stimuli between arterioles and venules^46^.

The results of Nesper et al. for the superficial plexus (increase from dark to light) and the present results in the radial peripapillary capillaries (increase from dark to light) seem to be in agreement. A similar effect of a stimulus on the superficial plexus and the radial peripapillary capillaries would be plausible since the activity of the retinal nerve fiber layer is downstream of the inner retina. However, this gives nuance to the basal principle of NVC, metabolic need leading to vascular adaptation.

In general, we have to be aware of the limitation of the OCTA technique itself and the long acquisition times of OCTA in comparison with e.g., laser Doppler techniques or retinal vessel analyzer, which give an instant measurement.

Furthermore, OCTA determines for every pixel a binary response of flow and non-flow, without a clear indication of velocity or increase in perfusion^47^. Thus, the minute increase in perfusion by neurovascular coupling might not result in a morphological change, large enough to be observed with OCTA. This, together with the vasoconstrictive effects of mydriasis induction, could explain the absence of similar effects in eyes in mydriasis.

Of note, the comparison of macular and optic disc vessel density data of eyes with neutral and mydriatic pupils after dark exposure reveals a highly significant difference in this population. Thus, mydriasis induction by tropicamide 0.5% and phenylephrine 2.5% seems to decrease vessel density at the macula and the optic nerve. Topical phenylephrine, through α1-adrenoreceptors, has been shown by laser doppler flowmetry to induce a decrease in blood velocity in retinal arteries^48^. Similarly, tropicamide, through the muscarinic acetylcholine receptors, decreases retinal capillary perfusion in healthy individuals^49^. Literature is not conclusive on this important issue. An earlier study by Brücher et al. found no difference in OCTA parameters (macula) between neutral pupils and mydriasis in an older patient cohort with macular degeneration (induced by a tropicamide 0,5% and neosynephrin 2,5% mixture), as did Hohberger in a young population (30 persons, macula and optic disc, 0,5% tropicamide, 5% phenylephrine)^29,50^. However, Cheng et al. found in a small cohort (8 young subjects, 0.5% tropicamide/0.5% phenylephrine mixture) a reduced vessel density in the peripapillary region, but not in the macular region, as did Villatoro et al. (26 participants, 2.5% phenylephrine/0.5% tropicamide)^51,52^. These studies seem to suggest, that the capillaries at the optic nerve are more susceptible to vasoconstrictive medication than those at the macula. Cheng et al. proposes this as an evolutionary advantage, where the central vision must be protected from vasoconstriction in a fight or flight response, whereas the peripheral vision is less of important in these moments. Villatoro et al. suggests that these findings are mere aberrations induced by changes in pupil size or, alternatively, proposes that these findings are within boundaries of intra-session variation of vessel density. An important limitation for our findings is that the population with neutral pupils is not the same as the population with mydriatic pupils. For future studies, caution is warranted not to mix vessel density data acquired in different pharmacologically induced pupil conditions.

This study has several limitations. Our study population consisted of healthy subjects of a relatively young and narrow age range. Not all factors known to influence OCTA measurements were recorded and considered. Blood pressure, heart rate, eye pressure and refraction were measured and found to be in acceptable ranges, other parameters as physical fitness and hematocrit were not assessed^53,54^.

Pupil dilation affects OCTA measurements. The present study did not find a significant difference in quality index scores between the groups with neutral and mydriatic pupils. A near-significant correlation between the macular quality index and VDsM in eyes with mydriatic pupils was observed. This suggests an interaction between quality index and OCTA measurements. The present study was not blinded. Both the OCTA operator and the image analyst were aware of whether a test person was dark- or light-adapted before the OCTA measurement and whether pupils were neutral or in mydriasis. The OCTA operator was highly experienced in both neutral and mydriatic pupils. The analyst was not blinded since no qualitative assessment of his part was asked outside of artefact control and segmentation check/correction.

No direct conclusions should be drawn for subjects with systemic or ocular pathologies, subjects of other age groups or OCTA systems using an alternative segmentation system. Aditionally, these results are only applicable on subjects with neutral pupils and pupils dilated with a single drop of a tropicamide 0.5% and neosynephrin 2.5% mixture. Other protocols for mydriasis induction may have different effects on the retinal vasculature and influence OCTA measurements^55^.

Rather than submitting the subjects to 4 cycles of dark and light adaptation (2 with neutral pupils, 2 in mydriasis), we opted, to improve compliance, thus for 2 groups (neutral and mydriatic pupils), undergoing each 2 cyles of dark and light adapation.

Also, the data distribution in the two groups was different. For this reason, the comparison of OCTA data between the two groups (neutral and mydriatic pupils) must be handled with care.

Pupil size can have an impact on vessel density measurements. In this study, we did not measure the pupil size to prioritize OCTA measurements and to be able to measure a maximal effect of early dark and light adaptation. Furthermore, we did not record the pupil size in neutral pupils and in mydriasis at baseline. Considering this parameter in future studies would be valuable^51^.

In conclusion, this study reveals significant changes in optic nerve OCTA parameters in eyes with neutral pupils. This was not found in macular OCTA parameters in eyes with neutral and mydriatic pupils, neither in optic disc OCTA parameters in eyes in mydriasis. This observation suggests that light conditions could be a possible factor influencing OCTA measurements. The basis of neurovascular coupling, metabolic need leading to vascular adaptation, does not seem consistent with our current findings. Our results lay emphasis on the complexity of regulation mechanisms in the different regions and layers of the macula and optic nerve. Additionally, these data warn for the effect of mydriatic drops on vessel density measurements. Further studies are needed to evaluate effects of alternative stimuli protocols, concentration, and compositions of mydriasis-inducing agents.

## Data Availability

The datasets generated during and/or analysed during the current study are available from the corresponding author on reasonable request.
